# Co-Occurrence, Predictors, and Related Aggressive Behaviors of Cognitive and Emotional Relative Deprivation Based on Latent Class Analysis

**DOI:** 10.3390/bs13070586

**Published:** 2023-07-13

**Authors:** Yunlong Tian, Haoyuan Zheng, Wei Tong, Wen He

**Affiliations:** 1 Department of Psychology, Shanghai Normal University, Shanghai 200234, China; 2 School of Teacher Education, Guangzhou Huashang College, Guangzhou 511300, China

**Keywords:** adolescent, relative deprivation, life satisfaction, individual difference, latent class analysis

## Abstract

Given the frequent occurrence of relative deprivation among adolescents and its negative effects, this study investigated relative deprivation among adolescents using a person-centered statistical technique (*n* = 1196; 565 girls). Latent class analysis identified three groups: low cognitive and emotional relative deprivation (Class 1, 33.78% of adolescents), high cognitive and low emotional relative deprivation (Class 2, 37.79% of adolescents), and high cognitive and emotional relative deprivation (Class 3, 28.43% of adolescents). Adolescents with low income and without parental accompaniment were more likely to be assigned to Classes 3 and 2. Compared with Class 1, Classes 3 and 2 had significant positive predictive effects on physical aggression, relational aggression, and overall aggressive behavior. The classes of relative deprivation influenced both physical and relational aggression, but not verbal aggression. Based on these findings, demographic characteristics and latent classes of relative deprivation should be considered together when developing interventions for aggressive behaviors.

## 1. Introduction

Income inequality in China has escalated due to the general economic growth since 1978 [1]. Individuals tend to compare themselves to those who are better off instead of worse off than themselves [2]. Exposure to such an unequal distribution of access to resources may lead to relative deprivation. Relative deprivation refers to an individual’s belief that he or she is disadvantaged compared with others—a feeling that arises from social comparison rather than objective economic or social status [3,4,5]. Adolescence is a critical phase for socialization, during which individuals actively seek to establish social connections and engage in increased social communication; during this time, they can experience enhanced perceptions of relative deprivation [6,7,8]. Strong relative deprivation can impair physical and mental health [9] and increases the risk for aggressive behaviors [4]. Therefore, it is of both practical and academic importance to investigate the causes and outcomes of relative deprivation. This study thus identified the patterns of relative deprivation among adolescents using latent class analysis (LCA), and explored relative deprivation’s risk factors and its effects on different aggressive behaviors based on LCA.

### 1.1. Cognitive and Emotional Relative Deprivation

Relative deprivation theory divides personal relative deprivation into two components: cognitive relative deprivation, referring to one’s perceptions of deprivation, and emotional relative deprivation, referring to one’s feelings about deprivation. The emotional component of relative deprivation is an essential element based on the cognitive component. However, the emotional component might not always align perfectly with the cognitive component [3]. For instance, prior work found that for impoverished villagers in Malawi, living near wealthy friends or neighbors does not lead to emotional relative derivation, such as envy or resentment [10]. Therefore, it is necessary to conduct research based on cognitive–emotional relative deprivation in order to explain personal characteristics and the influence based on relative deprivation theory. To date, no study has examined adolescent-specific patterns of relative deprivation based on its cognitive and emotional dimensions using relative deprivation theory. This literature gap is significant because relative deprivation is generated by cognitive evaluations that lead individuals to perceive themselves as disadvantaged [3]. That is, cognitive relative deprivation represents the initial source of relative deprivation. However, emotional relative deprivation is thought to capture the core of relative deprivation [11]. Therefore, using person-centered statistical analysis approaches to identify relative deprivation patterns based on cognitive and emotional components could provide a more nuanced picture of relative deprivation.

This study used LCA, a person-centered statistical method, to identify patterns in the cognitive and emotional dimensions of relative deprivation among adolescents and their relationship with diverse aggressive behaviors. LCA estimates class sizes based on a specific pattern of category probabilities for each item and assigns individuals to latent classes based on the highest membership probability. This analysis method has been widely used to identify latent groups in developmental psychology research because it enables the identification of specific patterns and alternative explanations for quantitative constructs [12,13]. We used the observed indicators and individuals’ responses of relative deprivation as characteristics to assign individuals to various classes through LCA. To the best of our knowledge, only Osborne et al. [14] focused on individual and group relative deprivation patterns using LCA, but did not specifically examine the unique patterns of cognitive and emotional relative deprivation. Our study thus aimed to fill this gap by investigating individual patterns of adolescent relative deprivation, specifically focusing on its cognitive and emotional dimensions.

### 1.2. Predictors of Relative Deprivation

Relative deprivation results from an individual’s subjective perception that she or he is in a disadvantaged situation based on upward social comparison with others in similar situations. Considering the high signification of peers during adolescence, individuals may unintentionally compare themselves to other adolescents [15]. Family income is an objective resource that could offer a high-quality life to adolescents. Prior work indicated that individuals may instinctively seek out basic resources, such as health and housing, and also secondary resources, such as SES, to acquire or protect the basic resources [16]. Therefore, faced with the objective reality of the higher income and intact family environment of other adolescents, when adolescents make upward social comparisons, they likely perceive their status to be low or disadvantaged; thus, some adolescents likely experience psychologically subjective “relative deprivation.” Indeed, prior work found that parents’ economic status is associated with relative deprivation levels in adolescents. In addition, parental accompaniment may affect adolescents’ relative deprivation. This is because parents are the main sources of adolescents’ relevance and belonging needs [17,18]. However, some Chinese parents often leave their adolescent children at home with only one parent or with their grandparents due to work obligations, resulting in their need for belonging and connection not being met. Such unmet needs may in turn lead teenagers to believe that they lack resources and, thus, elicit relative deprivation.

Finally, we proposed that gender may also play a significant role in relative deprivation. As the country is rooted in patriarchal traditions, for Chinese men, the importance of pursuing a career and taking responsibility for supporting a family has been historically underscored [19]. For instance, prior studies found that the main pressure on traditional China’s young people comes from their annual family income. In such an environment, males, even adolescent ones, are sensitive to objective resources and can easily make upward comparisons. An increasing body of empirical work supported this idea. For instance, Zhang and Tao found that male college students felt stronger relative deprivation than female students [20]. As such, we assumed that gender, parental accompaniment, and monthly family income are associated with adolescents’ assignment to specific latent classes of relative deprivation. Understanding the potential effects of these demographic factors on the generation of relative deprivation can help guide interventions for at-risk adolescents. By identifying those more likely to experience relative deprivation, interventions can meet adolescents’ specific objective and subjective needs.

### 1.3. Relative Deprivation and Aggressive Behavior

The prevalence of Chinese adolescents witnessing or experiencing violence has been reported to be 33.8% and 41.7%, respectively [6], making aggressive adolescent behavior a pressing public concern. There is evidence that relative deprivation contributes to the development of aggressive behavior [21,22]. According to the theory of relative deprivation, an individual’s objective position in a social hierarchy evokes a comparison between the individual and other similar people. If the individual perceives that other people are better off and that this relatively disadvantaged situation is unfair, he/she may respond with anger and resentment, which will in turn lead to aggressive behavior [23]. It is well known that Chinese adolescents experience intense social competition, which may instigate them to compare themselves with other adolescents intentionally or unintentionally. When they regard a negative situation as unfair, they may experience affective hostility and exhibit aggressive behaviors. For instance, Greitemeyer and Sagioglou found that relative deprivation is positively related to subsequent aggressive behavior [24]. Nieuwenhuis et al. found that when moving to a more affluent neighborhood, adolescents felt more relative deprivation and displayed more aggressive behaviors [25].

Despite previous research having established a link between relative deprivation and an increased risk of aggressive behavior among adolescents, different types of aggression (e.g., physical, verbal, relational) might have distinct causes. Hahn et al. [26], for instance, determined that verbal and physical aggression might be predicted by different factors and involve distinct mechanisms [23]. Additionally, relational aggression might be associated with an emotional component [27]. Such findings suggest that different patterns of relative deprivation can lead to different types of aggressive behavior. Given the distinct characteristics of the cognitive and emotional dimensions of relative deprivation, it is essential to explore the specific effects of relative deprivation.

To the best of our knowledge, this study is the first to investigate the relationship between the patterns of the cognitive–emotional dimensions of relative deprivation. This approach allows us to identify relative deprivation subgroups and their relationships with diverse aggressive behaviors and can inform intervention strategies to promote healthy development and prevent negative consequences. Therefore, this study aimed to support related research by using LCA to identify the effects of relative deprivation subgroups on different aggressive behaviors among adolescents.

### 1.4. The Present Study

This study used LCA to identify relative deprivation subgroups. Furthermore, we examined the relationships between predictor variables and these subgroups in adolescents. Finally, multiple regression techniques were used to test the specificity of relative deprivation’s effects on various types of aggressive behavior. Accordingly, this study contributes to developing interventions to promote healthy adolescent development and prevent the negative consequences of relative deprivation.

## 2. Methods

### 2.1. Participants

We used the random cluster approach to recruit participants. Questionnaires were anonymized to encourage honest reporting and avoid social desirability bias. A sample of 1196 adolescents (565 girls; *M* age = 14.75 years, *SD* = 1.69) from six senior and high schools in two provinces of China responded to the anonymous questionnaire. Written informed consent was obtained from the adolescent participants’ teachers and parents/legal guardians before the survey. The study was conducted according to the guidelines of the Declaration of Helsinki and approved by the Ethics Committee of Shanghai Normal University (protocol code 076 on 6 November 2022). Ethics approval was obtained from the authors’ affiliated institution. In the sample, 46.31% of the participants declared a monthly per capita family income of ¥0–3000, and 12.88% were above ¥8000. According to the participants, 90.8% of their fathers and 87.0% of their mothers had an educational level above senior middle school. Considering that some parents work in areas other than where their homes are, 7.83% of participants reported that they lived with both parents, while 14.52% lived with only one parent.

### 2.2. Measures

#### 2.2.1. Relative Deprivation

Relative deprivation was measured using the Adolescents’ Relative Deprivation Scale [28]. It includes 10 items and two subscales: cognitive (items 1–5) and emotional (items 6–10) relative deprivation related to social comparison in various aspects of life, such as friendship and family economic situation. Items are measured on a five-point Likert scale (1 = *strongly disagree* to 5 = *strongly agree*). Example items include “Compared with my friends, my parents are generally less supportive of me than their parents are of them” and “I’m frustrated by my pocket money relative to my friends”. We used the average scores for these items to assess relative deprivation levels; higher scores indicated higher levels of relative deprivation. In this study, the Cronbach’s αs were 0.807 (cognitive subscale), 0.867 (emotional subscale), and 0.879 (full scale).

#### 2.2.2. Aggressive Behavior

Aggressive behavior was measured using the 19-item Chinese version of the aggression questionnaire [29]. The Chinese version was tested and shown to be a good, reliable, and valid measurement tool [6,30]. It includes three subscales: physical aggression (eight items), verbal aggression (five items), and rational aggression (six items). Items are measured on a five-point Likert scale (1 = *not at all* to 5 = *absolutely like me*). The average score of the five items was used to assess life satisfaction, and higher scores mean higher life satisfaction. In this study, Cronbach’s αs for the subscales (physical, verbal, and rational aggression) and the whole scale were 0.79, 0.51, 0.66, and 0.84, respectively.

### 2.3. Analysis Plan

We used SPSS 26.0 to summarize the descriptive statistics and explore the associations between the study variables. Then, we used latent profile analysis to identify patterns of relative deprivation among adolescents in Mplus 7.4 [31]. A series of latent profile analysis models with various numbers of profiles were conducted to explore the number and structure of profiles. To select the optimal number of profiles, several model fit indices of these models were compared, including the Akaike information criteria (AIC), Bayesian information criteria (BIC), the significance of the Lo–Mendell–Rubin likelihood ratio test (LMRT) and the bootstrap likelihood ratio test (BLRT), and entropy. Specifically, low AIC and BIC values indicate a better fit; significant LMRT and BLRT values indicate that the k-profile model is significantly improved compared with the k-1-profile model; and entropy values greater than 0.8 indicate an adequate separation among profiles. These multiple criteria helped to identify explanations in the data for multiple class solutions. In addition, the final model selection was also conducted based on substantive theory and class sizes to ensure that profiles are meaningful, stable, and not over-extracted [31,32].

To examine the relationships between demographic characteristics and latent classes of relative deprivation, we used the most likely class membership as an observed variable in a multinomial logistic regression in SPSS. Specifically, after choosing the reference class, dummy variables were created for each of the other classes, which were then allowed to simultaneously examine various demographic variables. Finally, a multiple linear regression model was used to investigate the relationship between latent classes and aggressive behaviors.

## 3. Results

### 3.1. Model Fit Statistics and the Final Model

Table 1 presents the model fit statistics for the models with one to four latent classes. The AIC and BIC values decreased incrementally with the increase in the number of profiles, suggesting that the model fit continued to improve with the increasing number of profiles. All models had comparatively high values of entropy (>0.84). The LMRT and BLRT values of the four-class model were insignificant, indicating a lack of significance with increases in the number of latent classes. Hence, the four-class model was excluded, and the models with two to three classes were further investigated. Moreover, the three-profile solution identified an additional profile that was qualitatively distinguishable from those of the two-profile solution. Therefore, the three-profile solution was selected for further analyses.

**Figure 1:**
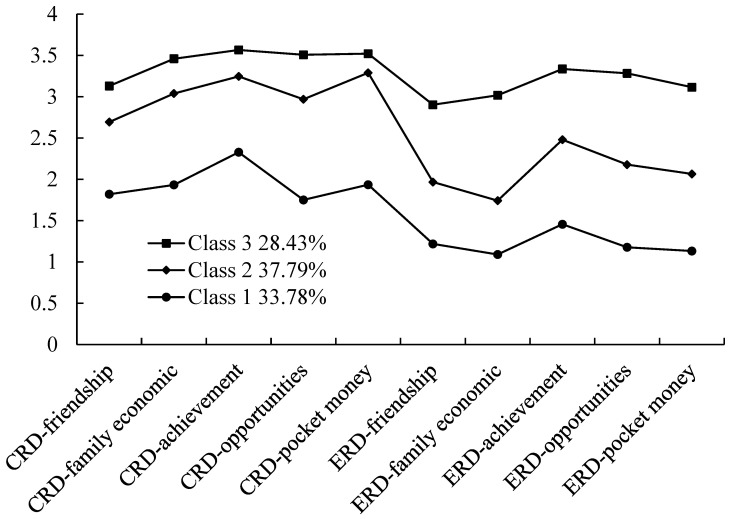
Expected means of items by latent class. Note: The *x*-axis shows the 10 items of relative deprivation; the *y*-axis shows the expected means for each class. CRD: cognitive relative deprivation; ERD: emotional relative deprivation.

Figure 1 illustrates the expected means for each class and item. LCA indicated that the differences were significant [F(2, 1193) = 1755.75, *p* < 0.001, *η*^2^ = 0.75]. Furthermore, each item in Class 1 (low cognitive and emotional relative deprivation) had lower scores than those in Class 2 (high cognitive and low emotional relative deprivation), and the scores for each item in Class 2 were lower than those in Class 3 (high cognitive and emotional relative deprivation). However, Class 2 had higher scores for cognitive relative deprivation than emotional relative deprivation.

### 3.2. Differences in Interclass Demographic Characteristics

We used multinomial logistic regression to test whether demographic characteristics affected latent class assignment. The data are shown in Table 2. Gender was non-significantly related to the probability of belonging to Classes 3 and 2, relative to Class 1. The probability of belonging to Classes 3 and 2, relative to Class 1, was lower for adolescents who lived with their parents compared with those who did not live with their parents. Finally, the probability of belonging to Class 3 relative to Class 2 was higher for adolescents with lower per capita monthly income than those with higher income.

### 3.3. Latent Classes and Aggressive Behavior

We performed multinomial linear regression using the mean whole scale and subscale scores for each class. The data are shown in Table 3. The latent classes of relative deprivation were the independent variables, and aggressive behaviors were the outcome variables. Latent classes were set as dummy variables since the independent variable was a class variable; the low cognitive and emotional relative deprivation class (Class 1) was the reference group. Compared with Class 1, Class 3 had positive predictive effects on physical aggression (*B* = 0.161, *p* < 0.001), relational aggression (*B* = 0.310, *p* < 0.001), and overall aggressive behavior (*B* = 0.166, *p* < 0.001). Compared with Class 1, Class 2 had positive predictive effects on physical aggression (*B* = 0.092, *p* = 0.033), relational aggression (*B* = 0.145, *p* < 0.001), and overall aggressive behavior (*B* = 0.108, *p* = 0.004). Furthermore, the predictive effects of Class 2 (*B* = 0.027, *p* = 0.546) and Class 3 (*B* = 0.007, *p* = 0.874) on verbal aggression were not significant.

## 4. Discussion

This study is the first to examine the latent classes of relative deprivation based on cognitive–emotion components and their effects on various aggressive behaviors. Our analyses of the participants’ response patterns led us to identify three latent classes. The analyses of demographic characteristics revealed possible risk factors for relative deprivation. The findings revealed that the three latent classes had significantly different aggressive behavior levels. These findings highlight the individual differences in adolescent relative deprivation and provide useful reference data for developing effective intervention strategies.

### 4.1. Latent Classes of Relative Deprivation

Adolescents in Class 1 had lower levels of both cognitive and emotional relative deprivation than those in other classes. Those in Class 3 had higher levels of both cognitive and emotional relative deprivation. Interestingly, in Class 2, which had high cognitive but low emotional relative deprivation, the scores for each item were in the middle compared with those of the adolescents in Classes 1 and 3. Relative deprivation theory proposes that the leading cause of relative deprivation is the belief that it is unfair for others to have a better social status than oneself [3,33]. Emotional relative deprivation can be lower than cognitive relative deprivation because individuals might accept their disadvantage as reasonable [3]. Thus, when adolescents perceive themselves as disadvantaged, they might not always view it as unfair, but instead accept their relative disadvantage. These adolescents might not experience strong emotional relative deprivation, but they might still experience cognitive relative deprivation [3,33].

The implications for interventions differ depending on the classes of relative deprivation. If the level of relative deprivation is moderate (Class 2), the interventions for cognitive strategies might be more efficient than those for emotional strategies. Thus, cognitive interventions could be developed to enhance the attentional filtering ability of Class 2 adolescents to reduce their cognitive relative deprivation [21]. However, if the relative deprivation level is high (Class 3), interventions for both cognitive and emotional strategies are warranted. For instance, it could be beneficial for interventions to focus on increasing social support empathy and reducing psychosocial vulnerability among Class 3 adolescents, which could help decrease their emotional relative deprivation [34,35].

### 4.2. Predictors of Relative Deprivation

We further explored the relationship between demographic characteristics and the classification of LCA. Objective status (e.g., economic status or clothing) provides a foundation for social comparison and can cause adolescents to assign themselves to a given relative position [36]. Some studies focused on relative deprivation among left-behind adolescents raised by their grandparents or a single parent [5,17,25,37]. We found that left-behind adolescents and those in low-income families had a high risk of relative deprivation. Thus, educators could pay attention to these adolescents and help guide their social actions to counter their experience of relative deprivation [5,34]. Notably, the relationship between gender and classes was non-significant. This can be understood in terms of the characteristics of adolescents [8]. Although female and male adolescents might focus on different aspects of life during this developmental period, both tend to frequently engage in interactions with others and make social comparisons. When we examined various aspects of life, no gender differences of relative deprivation were found. Therefore, stakeholders should aim to tackle relative deprivation among adolescents in both genders to promote their healthy development.

### 4.3. Relative Deprivation and Aggressive Behavior

Previous studies prove that relative deprivation can contribute to aggressive behavior [3,4,38,39]. Although studies suggest that different types of aggressive behavior might have different causes [4,24,40], the relationship between various characteristics based on the cognitive–emotional component of relative deprivation and different types of aggressive behavior remains unexplored. Based on the classes identified through LCA, our study supports the idea that different subgroups of cognitive–emotional relative deprivation can contribute to different types of aggressive behavior.

Compared with individuals in Class 1, those in Classes 3 and 2 were linked to high physical aggression, relational aggression, and overall aggressive behavior. Previous research suggests that physical and relational aggression have a consistent underlying motivation [41] and are associated with personal and social factors [42]. Consequently, it is likely that the cognitive and emotional components of relative deprivation are related to physical and relational aggression. Interestingly, the relationship between the latent classes of relative deprivation and verbal aggression was found to be non-significant. Previous studies suggest that verbal aggression is more strongly related to situational and interpersonal factors. The factors linked to relative deprivation (e.g., a lack of belonging and friendship) increase the risk of victimization in verbal aggression as opposed to perpetrating it against others [43,44,45]. Adolescents who perceive themselves as disadvantaged might display more physical and relational aggression owing to the negative perceptions and emotions related to relative deprivation, even if they have sufficient goods and resources.

### 4.4. Limitations and Future Research

This study has several limitations. First, the LCA results, based on observation data, are restricted to the characteristics of adolescents in China. Although existing research suggests that relative deprivation is a consistent predictor of individual development across different cultures, cultural differences are associated with the effects of relative deprivation [46]. Future studies could thus expand the sample to other countries and cultures for greater generalizability. Second, while this study did consider gender, income, and the presence of parents, it is essential to acknowledge the existence of additional factors that may influence patterns of relative deprivation. Finally, as our study design only allowed for the examination of correlational relationships, not causal ones, future research should investigate the causal relationships between relative deprivation and mental health outcomes.

Moreover, this study can help improve future research. Using our LCA-based findings as reference data to develop interventions could give researchers a more holistic account of individual differences in relative deprivation among adolescents. Furthermore, our findings based on demographic characteristics highlight the potential risk groups of relative deprivation among adolescents. We also explored how and toward whom the latent classes of relative deprivation affect physical, relational, and verbal aggression behavior. These findings offer valuable insights that can inform targeted recommendations for interventions aimed at addressing the adverse effects of different subgroups of relative deprivation.

## 5. Conclusions

Since the prevalence of relative deprivation among adolescents worldwide, identifying patterns of relative deprivation and examining their causes and outcomes could provide critical implications for theory and practice. In this study, we used LCA to identify three classes of relative deprivation based on cognitive and emotional relative deprivation: “high cognitive and emotional relative deprivation class” (Class 3), “high cognitive and low emotional relative deprivation” (Class 2), and “low cognitive and emotional relative deprivation class” (Class 1); this suggests that the heterogeneity of relative deprivation among adolescents exists. Moreover, the desynchronization of the cognitive and emotional relative deprivation class was observed within classes, supporting the notion that emotional relative deprivation does not always align with cognitive relative deprivation. Therefore, interventions aimed at decreasing adolescents’ relative deprivation should consider both cognitive and emotional relative deprivation. Interventions must be designed to target cognitive or emotional components.

In addition, we found that a lack of parental accompaniment and low family income can increase the risk in Classes 2 and 3, highlighting the influence of both subjective and objective resources on adolescents’ relative deprivation. Therefore, parents who leave their adolescents at home due to work obligations could use technological devices to keep in touch with adolescents and meet their belonging needs [47]. Furthermore, educators should pay more attention to adolescents from low-income families and provide social support, such as mental health assistance, to decrease relative deprivation among such adolescents.

Finally, previous research has examined the influence of relative deprivation on aggressive behaviors [23,24]. In this study, we further explored the relationship between classes of relative deprivation and different types of aggression. The results indicated that the latent classes of relative deprivation had an impact on both physical and relational aggressions. Thus, interventions designed for physical and relational aggression should consider the potential role of relative deprivation. By understanding these relationships, interventions can be refined to target the specific effects linking classes of relative deprivation and different types of aggression, ultimately leading to more successful outcomes in reducing aggressive behaviors among adolescents.

## Figures and Tables

**Table 1:** Results from the Latent Class Analytic Models.

Number of Classes	AIC	BIC	Entropy	LMRT (*p*)	BLRT (*p*)	Sample Size per Profile
1	36,532.138	36,633.872	-	-	-	1196
2	33,235.617	33,393.306	0.861	3276.489 ***	−18,246.069 ***	537, 659
3	32,443.485	32,657.128	0.848	803.821 **	−16,586.809 ***	404, 452, 340
4	31,922.406	32,192.004	0.851	536.200	0.1899	256, 402, 472,66

Note: AIC = Akaike Information Criterion; BIC = Bayesian Information Criterion. ** *p* < 0.01; *** *p* < 0.001.

**Table 2:** Results from the multinomial logistic regression evaluating the effects of demographic on latent classes membership.

		High Relative Deprivation (Class 3)		High Cognitive Relative Deprivation (Class 2)	
		OR	95% CI	OR	95% CI
Gender	Female	1.00		1.00	
	Male	1.31	0.978–1.745	1.23	0.930–1.635
Accompanied by parents	None	1.00		1.00	
	With father or mother	0.44 *	0.199–0.951	0.44 *	0.204–0.959
	With both parents	0.32 **	0.158–0.632	0.33 **	0.168–0.664
Per capita monthly income	More than ¥8000	1.00		1.00	
	¥3000~¥8000	2.37 **	1.442–3.883	1.96 **	1.225–3.133
	Less than ¥3000	4.01 ***	2.432–6.627	3.78 ***	2.352–6.061

Note: Reference group: low relative deprivation (Class 1); CI = confidence interval for the odds ratio; OR = odds ratio; * *p* < 0.05; ** *p* < 0.01; *** *p* < 0.001.

**Table 3:** Multiple linear regression model of the latent classes of relative deprivation with aggressive behavior and its dimensions as the dependent variables.

Latent Class	Aggressive Behavior		Physical Aggression		Relational Aggression		Verbal Aggression	
	*B*	95% CI	*B*	95% CI	*B*	95% CI	*B*	95% CI
Class 2	0.108 *	0.035–0.181	0.092 *	0.007–0.177	0.204 ***	0.110–0.298	0.027	−0.060–0.114
Class 3	0.166 ***	0.092–0.241	0.161 ***	0.074–0.248	0.310 ***	0.213–0.406	0.007	−0.082–0.096

Note: Class 2: high cognitive and low emotional relative deprivation; Class 3: high relative deprivation. Class 1: lower relative deprivation as dummy variables. * *p* < 0.05; *** *p* < 0.001.

## Data Availability

The data presented in this study are available on request from the corresponding author. The data are not publicly available due to privacy.
